# Preclinical optimization of an enterotoxigenic *Escherichia coli* adjuvanted subunit vaccine using response surface design of experiments

**DOI:** 10.1038/s41541-020-00228-w

**Published:** 2020-09-11

**Authors:** David Poncet, Catherine Hessler, Hong Liang, Sylviane Gautheron, Michelle Sergent, Nicholas D. Rintala, Emilie Seydoux, Po-Wei D. Huang, David Argilla, Sophie Ruiz, Jon Heinrichs, Milton Maciel, Mark T. Orr

**Affiliations:** 1 Sanofi Pasteur, Research and External Innovation, 1541 Av. Marcel Mérieux, 69280 Marcy L’Etoile, France; 2 Infectious Disease Research Institute (IDRI), 1616 Eastlake Ave E, Ste 400, Seattle, WA 98102 USA; 3 Aix Marseille Univ, Univ Avignon, CNRS, IRD, IMBE, Av. Escadrille Normandie Niémen, 13013 Marseille, France; 4 Sanofi Pasteur, 1 Discovery Dr, Swiftwater, PA 18370 USA; 5 Henry M. Jackson Foundation for the Advancement of Military Medicine, 6720A Rockledge Dr, Bethesda, MD 20817 USA; 6 Department of Microbiology and Immunology, Uniformed Services University of the Health Sciences, 4301 Jones Bridge Rd, Bethesda, MD 20814 USA; 7 Department of Global Health, University of Washington, 1510 San Juan Rd, Seattle, WA 98195 USA; 8 Present Address: Bristol Myers Squibb, Seattle, WA 98109 USA

**Keywords:** Adjuvants, Adjuvants

## Abstract

Enterotoxigenic *E. coli* (ETEC) is a leading cause of moderate-to-severe diarrhoea. ETEC colonizes the intestine through fimbrial tip adhesin colonization factors and produces heat-stable and/or heat-labile (LT) toxins, stimulating fluid and electrolyte release leading to watery diarrhoea. We reported that a vaccine containing recombinant colonization factor antigen (CfaEB) targeting fimbrial tip adhesin of the colonization factor antigen I (CFA/I) and an attenuated LT toxoid (dmLT) elicited mucosal and systemic immune responses against both targets. Additionally, the toll-like receptor 4 ligand second-generation lipid adjuvant (TLR4-SLA) induced a potent mucosal response, dependent on adjuvant formulation. However, a combination of vaccine components at their respective individual optimal doses may not achieve the optimal immune profile. We studied a subunit ETEC vaccine prototype in mice using a response surface design of experiments (DoE), consisting of 64 vaccine dose-combinations of CfaEB, dmLT and SLA in four formulations (aqueous, aluminium oxyhydroxide, squalene-in-water stable nanoemulsion [SE] or liposomes containing the saponin Quillaja saponaria-21 [LSQ]). Nine readouts focusing on antibody functionality and plasma cell response were selected to profile the immune response of parenterally administered ETEC vaccine prototype. The data were integrated in a model to identify the optimal dosage of each vaccine component and best formulation. Compared to maximal doses used in mouse models (10 µg CfaEB, 1 µg dmLT and 5 µg SLA), a reduction in the vaccine components up to 37%, 60% and 88% for CfaEB, dmLT and SLA, respectively, maintained or even maximized immune responses, with SE and LSQ the best formulations. The DoE approach can help determine the best vaccine composition with a limited number of experiments and may accelerate development of multi-antigen/component ETEC vaccines.

## Introduction

Enterotoxigenic *Escherichia coli* (ETEC) is the eighth leading cause of diarrhoea-related mortality worldwide^1^. It is a major cause of diarrhoea in developing countries where there is inadequate access to clean water and poor sanitation^2^, and among travellers and military personnel deployed in Africa, Asia and Latin America^3,4^. Despite a decrease in diarrhoea-related mortality since 1990, there were an estimated 18,669 deaths among children aged younger than 5 years and 51,186 deaths in all-age groups in 2016^1^.

ETEC, a rod-shaped Gram-negative bacterium, colonizes the human small intestine through chromosomal and/or plasmid-encoded fimbrial colonization factors (CFs) or coli surface antigens binding to enterocytes in the upper small intestine. The bacterium also produces heat-stable toxins (ST) and/or heat-labile toxins (LT) that stimulate the release of fluid and electrolytes from the intestinal epithelium, resulting in watery diarrhoea^4–6^. Since ETEC causes noninvasive, gut-associated mucosal infections, the attachment step is critical for the bacteria to effectively deliver toxins responsible for symptoms. A potent local immune response that blocks adhesion and neutralizes toxins may play a major role in protective immunity and thus, represents a potential strategic target for preventing ETEC infection^7,8^.

CF/coli surface antigens have long been a primary target for vaccine research and development due to their putative role in conferring protective immunity^9–13^. Recently, passive immunization with hyperimmune bovine immunoglobulin G (IgG) raised against whole colonization factor antigen I (CFA/I), as well as the tip adhesin colonization factor antigen fimbrial subunit E was shown to reduce the attack rate of a CFA/I, LT^+^, ST^+^ expressing ETEC strain^14^. The addition of an LT component may help improve vaccine immunogenicity and vaccine strain coverage against LT-only strains that lack CF/coli surface antigens. Short-term protective efficacy against LT^+^ ETEC has been documented with the cholera vaccine, Dukoral^®^, due to the production of cross-reactive LT-specific antibodies^15–17^.

Although there is no established immune correlate of protection against ETEC, these observations suggest that an effective ETEC vaccine should elicit immunity to CFs and toxins, and achieve optimal and synergistic local response at the intestinal mucosa. A double-mutant attenuated form of LT (dmLT) has been developed, R192G/L211A dmLT, that has immunogenic and mucosal adjuvant properties with an acceptable safety profile when administrated orally or parenterally in various animal models, and orally to humans^5,18^. In the present studies, the target immune profile for a parenteral vaccine delivered by intramuscular (IM) route was thus defined based on three aspects of humoral immunity: (1) induction of antibodies with functional activity to either prevent binding of ETEC to mammalian cells or neutralize LT (Haemagglutination Inhibition assay [HAI], LT-induced cyclic adenosine monophosphate [cAMP] production inhibition [LT neutralization]); (2) antigen-specific antibody titres at the site of ETEC infection, the intestinal mucosa (enzyme-linked immunosorbent assay [ELISA]); and (3) bone-marrow resident antigen-specific antibody-secreting plasma cells [ASC] that are able to maintain a durable antibody response. Using these four assays, the immune response was assessed in either sera or intestinal washes (IW), or in bone marrow collected after the first or second IM administration, resulting in a total of nine immunological readouts (Table 1, column A).

**Table 1:** Immunological readouts (A) and desirability function parameters (B–D).

Y	A	B	C	D
	Immune response	Lower bounds (log_10_)	Upper bounds (log_10_)	Weighting
Y1	Day 21 serum HAI	2.17	3.31	4
Y2	Day 35 serum HAI	3.60	4.52	3
Y3	Day 35 IW HAI	2.17	3.01	5
Y4	Day 21 serum LT neutralization	1.33	3.86	4
Y5	Day 35 serum LT neutralization	2.98	5.32	3
Y6	Day 35 IW dmLT IgG	3.10	5.19	1
Y7	Day 35 IW CfaEB IgG	3.52	5.32	1
Y8	Bone marrow dmLT ASCs	1.09	2.42	2
Y9	Bone marrow CfaEB ASCs	1.97	3.00	2

The candidate vaccine used in the present study was composed of CfaEB, consisting of the minor and major subunits of the CFA/I fimbriae stabilized by cis-donor strand complementation^19^, and dmLT. Additionally, an adjuvant such as the synthetic Toll-like receptor 4 ligand (TLR4)-second generation lipid adjuvant (SLA), designed to optimize TLR4 engagement through modifications to the acyl chains^20^, can generate a potent immune response by enhancing the systemic and mucosal functional antibody responses against CFA/I and LT^21^. This benefit extends to increased serum LT-neutralizing antibody titres, serum and mucosal HAI titres and intestinal immunoglobulin A titres. The adjuvant activity of TLR4 ligands can also be modified by altering the formulation in which they are presented^22^. Specifically, we found that formulating otherwise insoluble TLR4 agonists, such as SLA or glucopyranosyl lipid adjuvant, in a micellar aqueous formulation (AF) to solubilize vaccine component is critical for adjuvant activity^23^. Formulation of SLA-AF on aluminium oxyhydroxide (Al) augments the adjuvant’s potential to boost humoral immunity. Changing the formulation to a squalene-in-water stable nanoemulsion (SE) or in liposomes containing the saponin Quillaja saponaria-21 (LSQ) further enhances the adjuvant’s capacity to elicit robust cellular and humoral immunity^22,24,25^.

Antigen and adjuvant dosing may have a significant impact on the immunogenicity, efficacy and safety of a multi-component candidate vaccine consisting of CfaEB, dmLT, and SLA. We hypothesized that a vaccine based on the combination of the components at their respective individual optimal doses may not achieve the desired immune profile, due to possible interactions between the different components. Therefore, we used a design of experiments (DoE) approach based on response surface methodology to optimize CfaEB, dmLT and SLA doses in four different formulations (SE, LSQ, Al and AF). Moreover, a desirability function allowed us to consider all nine assessed immune responses in a unique mathematical model^26^, to identify the best target immunological profile^27^. These approaches help determine the best vaccine composition from a limited number of experiments^28–30^.

## Results

### Validation of the mathematical models

The immune response induced by the 64 tested vaccine combinations was assessed by measuring Day 21 and Day 35 serum HAI, Day 35 IW HAI, Day 21 and Day 35 serum LT-neutralization, Day 35 IW anti-dmLT and anti-CfaEB IgG, and dmLT and CfaEB bone marrow ASCs (Table 1 and Supplementary Table 1). As a first step, the postulated mathematical model was applied to the nine readouts to model the response across the 14 groups per formulation. Results generated at this first step were depicted for four immune responses of the SE formulation arbitrarily selected based on the representativeness of their mathematical interpretation and without considering the weighting (Day 21 serum HAI titres, Day 35 serum LT neutralization titres, Day 35 IW dmLT-specific IgG titres, and Day 35 bone-marrow resident CfaEB-specific ASC). Once the mathematical models were statistically validated, they were integrated into a multicriteria optimization using the desirability function approach to determine the ‘compromise zone’ where all experimental responses were satisfactory.

Finally, the mathematical predicted optimal vaccine compositions were confirmed in an additional mouse study.

### CfaEB dose has the major impact on Day 21 serum HAI titres

**Fig. 1:**
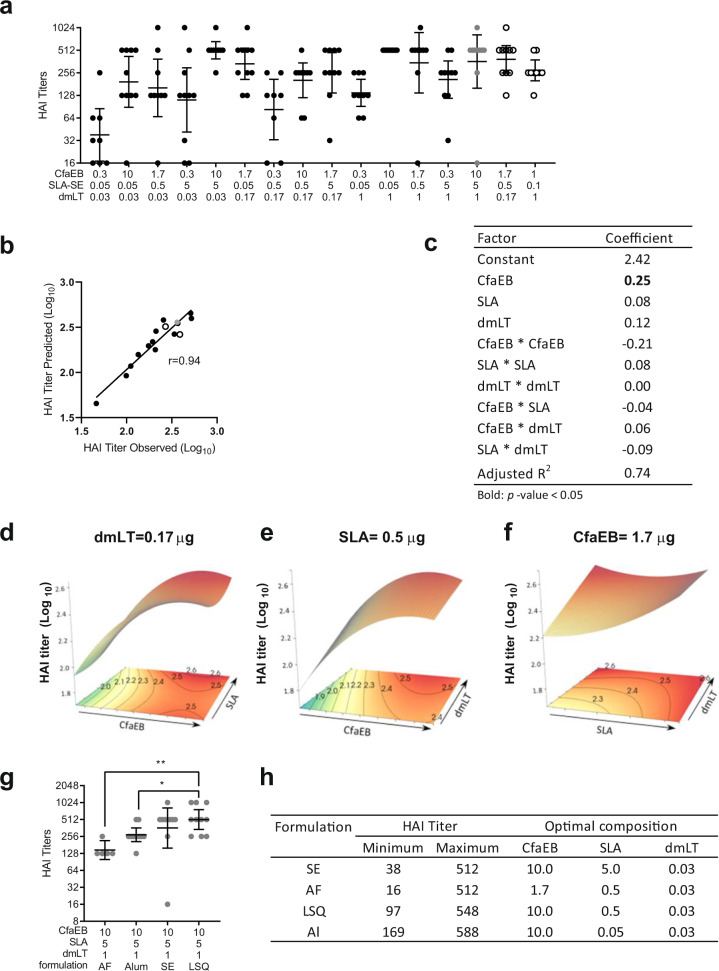
Day 21 serum HAI titres. BALB/c mice (*n* = 10 per group in two separate experiments) were immunized via IM injection with CfaEB, dmLT, and SLA formulated in SE. **a** Serum was collected on Day 21 and HAI conducted with human red blood cells using ETEC strain H10407. **b** The 14 point model based on the black and grey points accurately predicts the HAI titres observed for the two test points indicated in open circles. The non-parametric Spearman correlation coefficient *r* is shown. **c** A 10 component model was fitted to the 16 observed data points incorporating main, curvature, and pairwise interaction effects. Surface responses are shown as a function of **d** CfaEB vs. SLA at 0.17 µg dmLT, **e** CfaEB vs. dmLT at 0.5 µg SLA, **f** SLA vs. dmLT at 1.7 µg CfaEB. **g** HAI titres were determined as in **a** for animals immunized with the LSQ, AF or Al formulations and the titres for the maximal dose vaccines for each formulation are shown. **h** The observed range of HAI responses and dose composition (µg) for each optimal vaccine composition are shown by formulation. Grey data points indicate the maximal dose vaccine composition. Open circles indicate the two test vaccine compositions. Lines represent geometric means and whiskers indicate the 95% confidence intervals. Statistical analysis was performed with Student’s *T* test, or one-way ANOVA with Tukey’s multiple comparisons post-test (two-sided). *p < 0.05; **p < 0.01.

The capacity of the serum antibodies to prevent ETEC strain H10407, expressing CFA/I, from agglutinating red blood cells as a surrogate measurement for prevention of intestinal colonization was assessed 3 weeks after the priming IM immunization in mice (Fig. 1a). The experimental composition with the highest doses of the CfaEB and dmLT antigens, and SLA-SE adjuvant did not produce the highest HAI titre (Fig. 1a). This reinforces the necessity of determining the impact of each component dose on the immune response. The 14 groups, excluding the two test points shown in open circles, were used to build a mathematical model to estimate the HAI titre as a function of the doses of the three components in the SE formulation. This model, which incorporated the main effects of the three components, the curvature effects of each component, and the pairwise interactions between each component, closely fitted the experimental data (non-parametric Spearman correlation *r* = 0.94) (Fig. 1b). The predictive power of this model was quite robust within the bounds of the doses tested as the observed HAI titres for the two test points closely matched the values predicted by the model (Fig. 1b). We developed a slightly revised predictive model by also incorporating the two test points to increase the power of the model (adjusted *R*^2^ = 0.74). In this revised model, the dose of the CfaEB component, as expected, had the most significant impact on the predicted HAI titre (CfaEB: linear and squared coefficients 0.25 and –0.21, respectively, Fig. 1c). We observed a predominant influence of the CfaEB concentration on the HAI titre (Fig. 1d, e) in the response surface graphs, whereas for a fixed concentration of CfaEB, the variation of the response was very low (Fig. 1f).

At the maximal doses, changing the SLA formulation from SE to the simpler AF formulation reduced the HAI titre after the priming immunization when the other vaccine components were held constant. Compared to the SE formulation there was a trend towards higher HAI titres with the LSQ formulation, and towards lower titres with the Al formulation (Fig. 1g). The change in formulation also altered the impact of varying the doses of dmLT, CfaEB and SLA on the HAI response (Supplementary Table 1). For the SE, LSQ, and Al formulations, the HAI titre was maximized at the highest dose of CfaEB, whereas the intermediate dose of CfaEB was optimal for the AF formulation (Fig. 1h). In all four formulations, minimizing the dmLT dose produced the highest HAI titres. Interestingly, the optimal dose of SLA varied with each of the formulations; the maximal dose of SLA was required for the highest HAI titres in the SE formulation, the intermediate dose was optimal for both the AF and LSQ formulation, and the minimal SLA dose produced the highest HAI titres in the Al formulation (Fig. 1h).

There was an overall increase in serum HAI titres 2 weeks after the booster. Moreover, the impact of the vaccine dose components and formulation on these responses were different from those on Day 21 post-prime time point in serum (Supplementary Table 1).

### SLA enhanced LT neutralizing antibodies elicited by dmLT

**Fig. 2:**
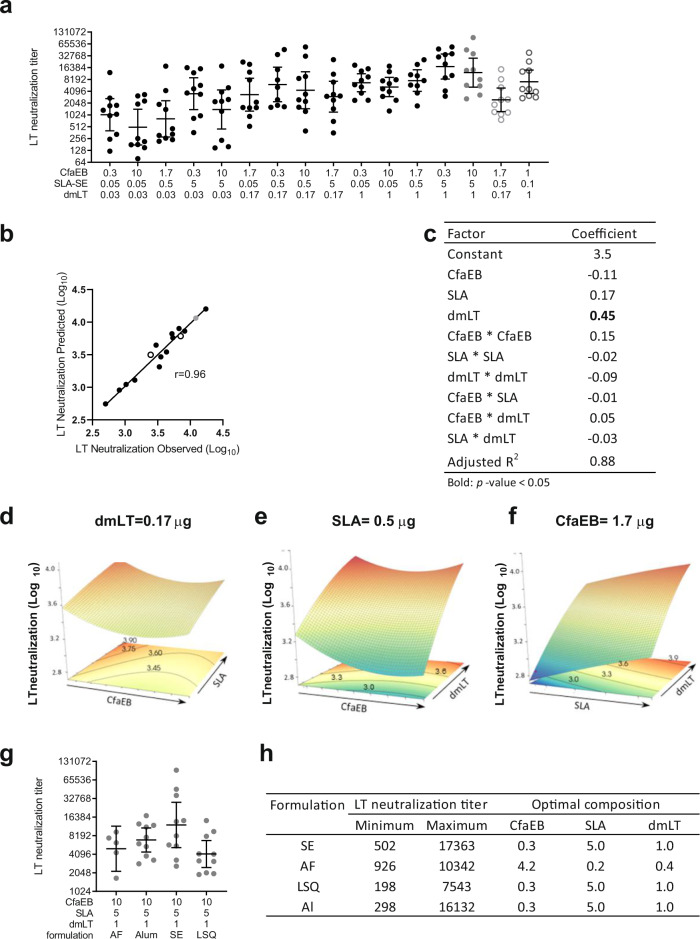
Day 35 serum LT neutralization titres. BALB/c mice (*n* = 10 per group in two separate experiments) were immunized via IM injection with CfaEB, dmLT, and SLA formulated in SE with a repeat booster injection on Day 21. **a** Serum was collected on Day 35 and functional LT neutralizing titres were determined by inhibition of cAMP flux in Vero cells treated with LT. **b** The 14 point model based on the black and grey points accurately predicts the LT neutralization titres observed for the two test points indicated by open circles. The non-parametric Spearman correlation coefficient *r* is shown. **c** A 10 component model was fitted to the 16 observed data points incorporating main, curvature and pairwise interaction effects. Surface responses are shown as a function of **d** CfaEB vs. SLA at 0.17 µg dmLT, **e** CfaEB vs. dmLT at 0.5 µg SLA, **f** SLA vs. dmLT at 1.7 µg CfaEB. **g** LT neutralization titres were determined as in **a** for animals immunized with the LSQ, AF or Al formulations and the titres for the maximal dose vaccines for each formulation are shown. **h** The observed range of LT neutralization responses and dose composition (µg) for each optimal vaccine composition are shown by formulation. Grey data points indicate the maximal dose vaccine composition. Open circles indicate the two test vaccine compositions. Lines represent geometric means and whiskers indicate the 95% confidence intervals. Statistical analysis was performed with Student’s *T* test.

LT is a hetero-hexameric A-B_5_ toxin, in which the B subunit binds to the cell surface whereas the A subunit is cleaved and translocated into the cell, where activation of G protein Gsα by LTA1 leads to irreversible activation of adenylate cyclase. This in turn causes rapid production of cAMP, which activates the cystic fibrosis transmembrane conductance regulator channel to secrete Cl^–^^31^. As such, inhibition of cAMP flux in response to LT toxin exposure is indicative of toxin-neutralization. Neutralization of LT toxicity may prevent the binding of the B_5_ pentamer or the enzymatic activity of the A subunit, either of which would consequently prevent cAMP flux in target cells^32^. Thus, we used inhibition of LT-induced cAMP flux as an assay to measure LT-neutralizing antibody titres. Two weeks after the booster immunization there was a substantial increase in LT-neutralizing antibody titres in the serum of mice immunized with the SE formulation (Fig. 2a). The resultant model produced a strong correlation (non-parametric Spearman correlation *r* = 0.96) between the observed and predicted LT neutralization values (Fig. 2b). The model showed good predictive power with the observed values of the test groups closely matching those predicted by the model (Fig. 2b, open circles) (adjusted *R*^2^ = 0.88). The dose of dmLT, as expected, had the greatest influence on LT-neutralizing titres (dmLT linear coefficient 0.45, Fig. 2c, e, f), which was further enhanced with the inclusion of SLA. Surprisingly, the dose of CfaEB had a modest negative impact on the LT-neutralization titre (Fig. 2c–e).

Changing the formulation resulted in modestly lower LT-neutralizing titres with a vaccine composition of 10 µg CfaEB, 5 µg SLA, and 1 µg dmLT (Fig. 2g). The optimal vaccine composition for the SE, LSQ, and Al formulations all consisted of minimizing the CfaEB and maximizing the dmLT and SLA doses (Fig. 2h). As with the HAI titres, the impact of the vaccine composition on the levels of LT-neutralizing antibody was differentially impacted as vaccine formulation varied (Supplementary Table 2). On Day 21 post-prime, responses were lower compared to the post-boost response (mean titre 1.86 versus 3.50, respectively) but were still mostly influenced by the dmLT dose. Unfortunately, in this experimental study design, the LT neutralizing titres in the intestinal samples were below the level of quantitation.

### Increasing doses of dmLT and SLA produced stronger intestinal antibody responses

**Fig. 3:**
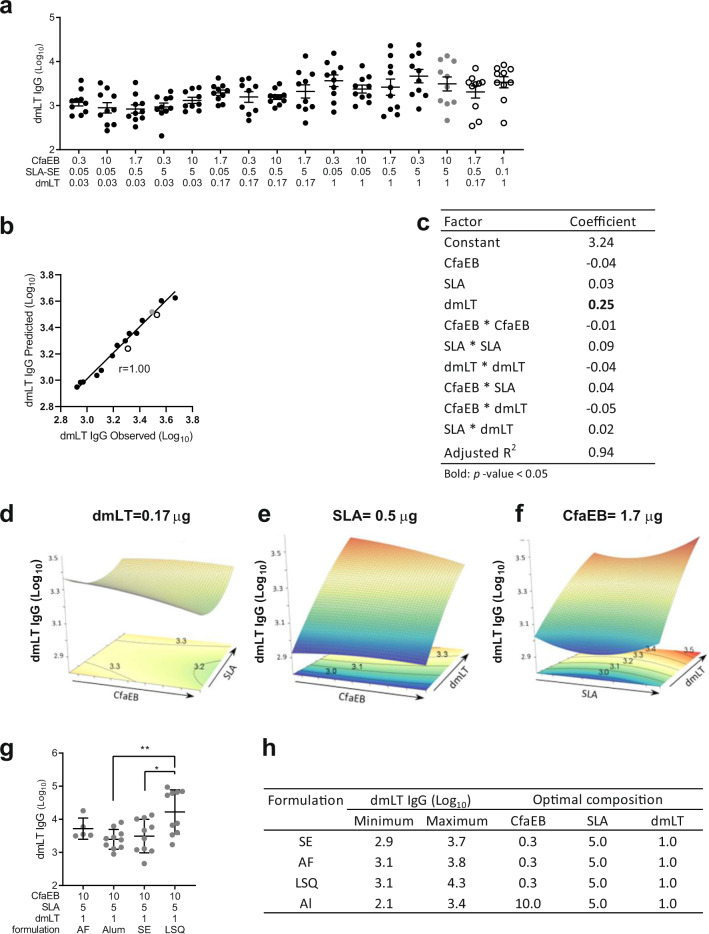
Day 35 intestinal wash dmLT-specific IgG titres. BALB/c mice (*n* = 10 per group in two separate experiments) were immunized via IM injection with CfaEB, dmLT, and SLA formulated in SE. **a** The ileum was washed with Hanks’ balanced salt solution at Day 35 to collect intestinal antibodies which were quantified by ELISA binding to dmLT. **b** The 14 point model based on the black and grey points accurately predicts the dmLT IgG titres observed for the two test points indicated by open circles. The non-parametric Spearman correlation coefficient *r* is shown. **c** A 10 component model was fitted to the 16 observed data points incorporating main, curvature and pairwise interaction effects. Surface responses are shown as a function of **d** CfaEB vs. SLA at 0.17 µg dmLT, **e** CfaEB vs. dmLT at 0.5 µg SLA, **f** SLA vs. dmLT at 1.7 µg CfaEB. **g** dmLT IgG titres were determined as in **a** for animals immunized with the LSQ, AF or Al formulations and the titres for the maximal dose vaccines for each formulation are shown. **h** The observed range of dmLT IgG responses and dose composition (µg) for each optimal vaccine composition are shown by formulation. Grey data points indicate the maximal dose vaccine composition. Open circles indicate the two test vaccine compositions. Lines represent geometric means and whiskers indicate the 95% confidence intervals. Statistical analysis was performed with Student’s *T* test, or one-way ANOVA with Tukey’s multiple comparisons post-test (two-sided). *p < 0.05; **p < 0.01.

Although the LT-neutralizing titres in the IW samples were below the limit of quantitation, IM immunization generated a high level of LT-specific antibodies in the intestine as detected by ELISA (Fig. 3a). The resultant model of the data fitted the observed dmLT IgG titres well (non-parametric Spearman correlation *r* = 1.00 between predicted and observed) and had a strong predictive capacity (Adjusted *R*^2^ = 0.94) as demonstrated by the close match of the observed and predicted titres for the two test points (Fig. 3b, open circles). Only the dose of dmLT had a significant impact on the responses (dmLT linear coefficient 0.25, Fig. 3c, e, f). In agreement with our previous findings^21^, the dose of SLA also had a modest impact as illustrated with the slight curvature effect on the dmLT IgG response (SLA × SLA coefficient of 0.09) (Fig. 3c, d, f).

Altering the vaccine formulation from SE to LSQ produced a significantly higher titre with the maximal dose of all three antigen and adjuvant components (Fig. 3g). For all four formulations, the strongest responses were observed when dmLT and SLA were maximized, with the minimal CfaEB dose being beneficial to the SE, AF and LSQ formulations (Fig. 3h). CfaEB-specific IgG were also detected in the IW and the titres were significantly enhanced by SLA dose in AF formulation (SLA coefficient 0.2, Supplementary Table 3).

### The choice of the formulation determines how SLA affects the number of CfaEB-specific antibody-secreting cells

**Fig. 4:**
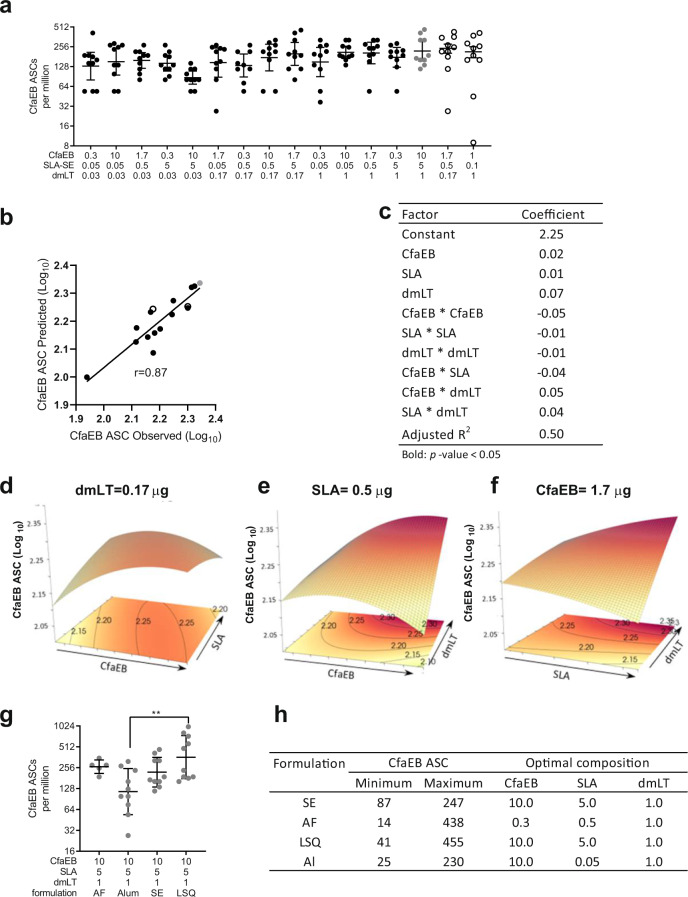
Day 35 bone-marrow resident CfaEB-specific antibody-secreting plasma cells. BALB/c mice (*n* = 10 per group in two separate experiments) were immunized via IM injection with CfaEB, dmLT, and SLA formulated in SE. Bone marrow was isolated from both rear femurs on Day 35 and red blood cells were immediately lysed. **a** Cells were stimulated with CfaEB and assessed for secretion of IgG by ELIspot. **b** The 14 point model based on the black and grey points accurately predicts the CfaEB ASC titres observed for the two test points indicated by open circles. The non-parametric Spearman correlation coefficient *r* is shown. **c** A 10 component model was fitted to the 16 observed data points incorporating main, curvature and pairwise interaction effects. Surface responses are shown as a function of **d** CfaEB vs. SLA at 0.17 µg dmLT, **e** CfaEB vs. dmLT at 0.5 µg SLA, **f** SLA vs. dmLT at 1.7 µg CfaEB. **g** CfaEB ASC titres were determined as in **a** for animals immunized with the LSQ, AF or Al formulations and the titres for the maximal dose vaccines for each formulation are shown. **h** The observed range of dmLT IgG responses and dose composition (µg) for each optimal vaccine composition are shown by formulation. Grey data points indicate the maximal dose vaccine composition. Open circles indicate the two test vaccine compositions. Lines represent geometric means and whiskers indicate the 95% confidence intervals. Statistical analysis was performed with Student’s *T* test, or one-way ANOVA with Tukey’s multiple comparisons post-test (two-sided). **p < 0.01.

Durability of immunity is important for prophylactic vaccines. Serum antibody titres are maintained by the constant production of new antibodies from plasma cells that reside in the bone marrow^33^. Thus, the number of these ASCs is a useful proxy for the longevity of the antibody response. Overall, there was very little variation in the magnitude of the response across all doses tested (Fig. 4a) (non-parametric Spearman *r* = 0.87 between observed and predicted [Fig. 4b]). Despite this, a modest predictive model (Adjusted *R*^2^ = 0.50) could be derived that accurately predicted the responses elicited by the two-test groups (Fig. 4c), with a modest influence of dmLT dose (dmLT coefficient 0.07, Fig. 4c–f).

At the highest doses of CfaEB, dmLT and SLA, the LSQ formulation produced significantly more CfaEB-specific ASCs than the Al formulation (Fig. 4g). For all formulations except AF, maximizing the CfaEB dose also had a beneficial effect whereas the optimal dose of SLA varied with the formulation (Fig. 4h). In all four formulations, the maximal dmLT and CfaEB-specific ASCs were elicited by maximizing the dmLT dose (Supplementary Table 4).

### Integration of the composite biological data using multicriteria optimization led to the identification of a lead vaccine composition

**Fig. 5:**
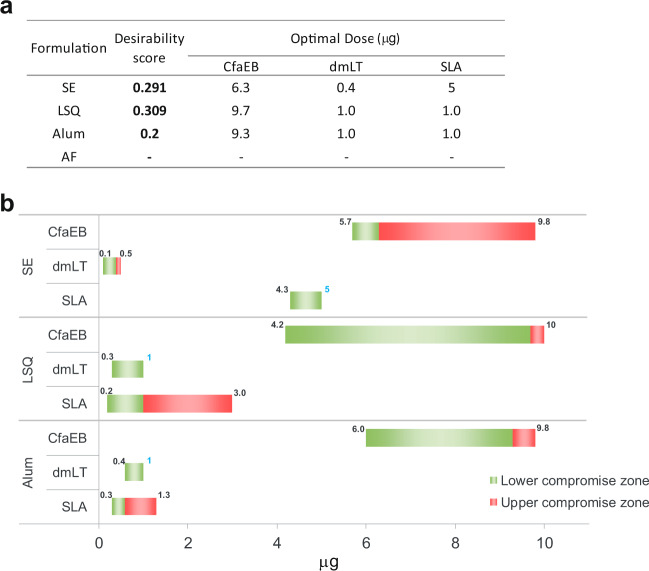
Global desirability. **a** Predicted optimized vaccine composition by formulation. **b** Representation of the compromise zone. The nine immunological parameters were integrated into the desirability function with the weighted importance. For each component of the vaccine, the zone around the optimum with satisfying desirability is shown (Nemrodw v2015). Numbers in blue indicate when upper compromise zone corresponds to the optimal.

Analysing individual immune responses can lead to distinct vaccine compositions. Consequently, the final selection of the vaccine composition should be a compromise that best solves the relative importance of each immune parameter (Table 2, illustrated for the SE formulation). All the mathematical models were considered in the desirability study, whether they were significant or not. According to the desirability score, the ranking of the formulations was LSQ, SE and AL, with no compromise zone enabled for AF (Fig. 5a).

**Table 2:** Optimal observed dose of each component for individual readouts with SE formulation.

	CfaEB (µg)	SLA (µg)	dmLT (µg)
Day 21 serum HAI	10	5	0.03
Day 35 serum HAI	10	5	0.03
Day 35 IW HAI	1.7	0.05	0.17
Day 21 serum LT neutralization	0.3	5	1
Day 35 serum LT neutralization	0.3	5	1
Day 35 IW dmLT IgG	0.3	5	1
Day 35 IW CfaEB IgG	1.7	0.05	0.17
Bone marrow dmLT ASCs	10	0.05	1
Bone marrow CfaEB ASCs	10	5	1

The predicted optimal vaccine composition and zone of compromise for 3 of the 4 formulations is shown in Fig. 5a, b, respectively. The optimal dose of CfaEB varied by about 50% (6.3–9.7 µg), depending on the formulation. The optimal SLA dose varied 5-fold, whereas the optimal dmLT dose only varied 2.5-fold. For the LSQ formulation, the compromise zone was very large, and to maximize the global desirability, CfaEB and dmLT must be high and SLA fixed at an intermediate value. The SE formulation needed a high concentration of SLA to satisfy the objectives, but the zone of compromise was larger for CfaEB and dmLT; while for the Al formulation, SLA can be fixed at an intermediate value but the CfaEB and dmLT concentrations must be high. For the AF formulation there was a region of incompatibility between several of the immune responses such that there was no minimally acceptable solution. This reflects the overall lower immune responses from the experimental groups with the AF formulation, particularly for the Day 35 IW and serum HAI responses, which did not meet the target minimum.

### Optimized SE and LSQ formulations of the CfaEB+dmLT+SLA vaccine induced better immune response than maximized dose formulations

To determine whether the predicted optimal vaccine compositions shown in Fig. 5 produced robust responses across the panel of immune parameters, we immunized cohorts of mice with the optimized vaccine doses in both the SE and LSQ formulations as well as the maximum control dose consisting of 10 μg CfaEB, 5 μg SLA, and 1 μg dmLT in SE formulation (referred to 10/5/1/SE in subsequent text). Control groups received CfaEB alone or CfaEB adjuvanted with only dmLT or SLA at doses used in the predicted optimal vaccines.

**Fig. 6:**
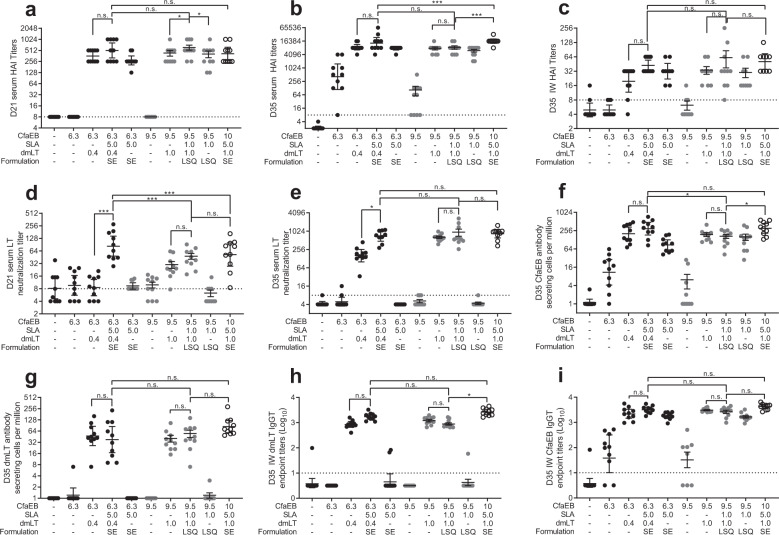
Immune responses to the predicted optimized formulations. BALB/c mice (*n* = 10 per group in two separate experiments) were immunized via IM injection with CfaEB, dmLT, and SLA formulated in SE or LSQ with a repeat booster on Day 21. Serum was collected on Day 21 and Day 35. IW samples and bone marrow cells were collected on Day 35. **a** Day 21 serum, **b** Day 35 serum, and **c** Day 35 IW serum functional anti-CFA/I antibody titres were determined by HAI with human red blood cells using ETEC strain H10407. **d** Day 21 and **e** Day 35 serum functional LT neutralizing titres were determined by inhibition of cAMP flux in Vero cells treated with LT. Day 35 bone marrow **f** CfaEB and **g** dmLT-specific ASCs were determined by ELISpot. Day 35 IW **h** dmLT and **i** CfaEB-specific IgG were determined by ELISA. Lines represent geometric means and whiskers indicate the standard deviations. Black circles, SE formulation; Grey circles, LSQ formulation; Open circles, 10/5/1 maximal dose control in SE formulation. Statistical analysis was performed with one-way ANOVA with Tukey’s multiple comparisons post-test (two-sided). n.s. not significant; *p < 0.05; ***p < 0.001.

Immunization with CfaEB alone did not produce detectable HAI antibodies in the post-prime serum or post-boost IW. Boosting with CfaEB generated a modest HAI titre in the serum, although this was improved with inclusion of SLA and/or dmLT (Fig. 6a–c). The optimized LSQ-formulated vaccine outperformed the 10/5/1/SE control for HAI generation in both the post-prime and post-boost serum. There was a trend that inclusion of both adjuvants was necessary to achieve the maximal HAI response, especially in the intestinal samples.

Generation of serum LT-neutralizing antibodies at Day 21 and Day 35 required inclusion of dmLT and were significantly augmented (*p* < 0.001 and *p* < 0.005, respectively) by the addition of SLA-SE adjuvant (Fig. 6d, e), in line with Liang et al^21^. After the priming immunization, the optimized vaccine induced significantly higher serum LT-neutralizing titres to the 10/5/1/SE reference group in the SE formulation (*p* < 0.001) and similar for LSQ (Fig. 6d). After the second immunization serum LT-neutralizing titres induced by optimal vaccine were similar to the reference group in both formulations (Fig. 6e). An adjuvant was needed for the generation of CfaEB-specific bone marrow ASCs with the inclusion of dmLT being more beneficial than SLA (Fig. 6f). The inclusion of SLA was not necessary to produce dmLT-specific bone marrow ASCs, suggesting that dmLT may be particularly effective in programming this type of immune response (Fig. 6g). Similarly, either adjuvant was sufficient to enhance intestinal IgG responses against CfaEB (Fig. 6i), which was not observed for dmLT (Fig. 6h). Taken together, these data show that the optimized SE and LSQ formulations of CfaEB+dmLT+SLA produce similar or superior immune responses to a composition that simply maximized all the doses.

## Discussion

Vaccine development requires consideration of different parameters, such as the composition and formulation of the vaccine, the animal model to perform preclinical testing, immunization schedule and route of administration. Selection of the most efficient route of immunization is one of the main challenges in vaccine development, especially to protect against infectious disease caused by a mucosal pathogen such as ETEC. In this context, mucosal delivery is attractive but, while oral route is commonly used for killed or live-attenuated vaccines^34,35^, it is not suitable for many subunit vaccines, since it will require a high dose of antigen to circumvent the risk of degradation in the stomach. Other mucosal routes, such as sublingual, are intensively studied^36,37^, but some limitations still need to be addressed^38^. Intradermal vaccine injection has been shown to elicit potent systemic and mucosal immune responses^39^, potentiate responses to co-administered antigens, as well as having antigen-sparing advantages^40,41^. However, in some cases, local adverse reactions seem to be more serious with intradermal than IM administration^42,43^. Our investigational products were administered intramuscularly to evaluate the more traditional vaccination route without need for costly devices or additional training, which would allow for more optimal transition for use in less developed countries. IM administration of vaccines has successfully been used for viral infections of the gastrointestinal tract such as norovirus and rotavirus^44–46^. Polio vaccine is equally effective given orally or by IM injections, and *Haemophilus influenzae* type b conjugate and pneumococcal vaccines administered by the IM route have been demonstrated to have an impact on respiratory carriage^47^.

Adjuvant selection is also a critical step for the development of effective subunit vaccines and the inclusion of Toll-like receptor agonists is an effective method of enhancing T cell responses elicited by vaccines^22^. The adjuvant activity of TLR4 ligands can also be modified by altering the formulation in which they are presented^20^. Furthermore, SLA-SE synergizes with dmLT to further augment both anti-CfaEB and anti-LT immune responses^21^. Our present study adds another aspect of vaccine optimization, that of vaccine component dosing in addition to the optimization of formulation. This is relevant for vaccine development as there is currently a lack of systematic robust rationale for vaccine component dosing, for both antigens and adjuvants. Optimizing each vaccine component can produce a more desirable immune response profile than simply maximizing the dose of each component and may potentially result in improved safety margin and reduced cost of the final vaccine.

Our study demonstrates the usefulness of the DoE approach in optimizing CfaEB, dmLT and SLA doses in four different formulations (SE, LSQ, Al and AF):

1. The optimal combination was determined from the mathematical model and thus may not be part of the conditions tested in the present DoE.
2. This strategy minimized the number of experiments. Whereas in a classical approach, 108 groups would have been needed (3 doses for CfaEB × 3 doses for SLA × 3 doses for dmLT × 4 formulations), we found that 64 groups were sufficient to model all nine surface responses with acceptable precision and accuracy. Consequently, this approach allowed for a reduction in the number of mice necessary for experimental optimization of the vaccine composition.
3. The desirability approach allowed integrating all nine readouts, targeting a desired hypothesized immunological profile and predicted an optimized vaccine composition by formulation based on the compromise zone for each component.

Our study helped determine that: (1) CfaEB had a major impact on HAI titres; (2) SLA enhanced LT-neutralizing antibodies elicited by dmLT; (3) increased doses of dmLT and SLA induced greater intestinal antibody responses; and (4) the formulation chosen determined how SLA affects the number of CfaEB-specific and dmLT-specific ASCs.

Importantly, we confirmed that, upon IM immunization, mice that received the optimized vaccine component doses from the mathematical models, in both SE and LSQ formulations, produced similar or superior immune responses to those of a composition that simply maximized all doses. These experiments showed that depending on the formulation, the optimal dose of CfaEB varied by about 50% and that of SLA varied almost 5-fold, whereas dmLT optimal dose varied 2.5-fold only. We found that a reduction in the vaccine components by up to 37% for CfaEB, 60% for dmLT and 80% for SLA in SE or LSQ formulations would be sufficient to produce immune responses similar or superior to the maximized-dose composition. Nevertheless, it is important to remember that the weighting of the immunological readouts has been defined in agreement with the current knowledge of potential vaccine efficacy against a mucosal pathogen. If soon, one of our readouts would appear as a surrogate of protection, the weighting could be updated accordingly, and the mathematical model might be realigned in accord with biological reality.

This preclinical work provides first insights on how these vaccine components can positively or negatively interact, helping vaccine developers to accelerate development of complex multi-antigen/component ETEC vaccines and ultimately save developmental costs by reducing cost of goods in the final formulation. In addition, this DoE approach coupled with an informed desirability function is amenable to considering additional parameters of vaccine optimization including the impact of sex on the optimal vaccine composition.

An effective ETEC vaccine should promote immunity against several CFs to allow for sufficiently broad coverage against a range of clinical strains^48^. The addition of dmLT to the formulation can elicit anti-LT immunity as well as enhance the response against different CF-based vaccine candidates, as has been recently demonstrated^38,49^. Using the same dose optimization approach, the next step will be to select the dose of other CF components, such as the recently described antigen coli surface antigen 6 fimbrial subunit B and A protein heterodimer, which elicits immunity to coli surface antigen 6^49^, to ensure immunity to all targeted CFs and also to avoid cross-antigen interference. These steps will benefit from the selection methods used in the present study and could be further leveraged by recent findings that anti-CfaEB IgG1 titres could predict anti-CF functionality measured by HAI, at least for class 5a antigens^38^. ST, a non-immunogenic peptide that is similar to the human peptides guanylin and uroguanylin could also be part of a future vaccine against ETEC and the dose could be optimized using the same approach. However, further studies are needed to advance candidate toxoids by ascertaining their ability to protect animals from ETEC diarrhoea against ST-only expressing strains with no risk of auto-immune disease^50^.

Although our approach is fully aligned with the principles of the 3Rs (Replacement, Reduction and Refinement)^51^, a large number of animals was still required in order to assess the many parameters and formulations with acceptable precision and accuracy. Such large studies cannot be done in non-human primates. In addition, there is a current lack of appropriate assays to assess all parameters. Our study was intended to be an initial first step in identifying the optimal vaccine composition with the ‘best’ target immunological profile that would then be taken forward for further assessment in other animal studies including non-human primates. The New World primate, *Aotus nancymaee*, has been shown to have several advantages over other animal models in assessing the immunogenicity and efficacy of subunit ETEC vaccines^19,52^, and more closely approximates human disease.

## Methods

### Design of experiments

The DoE was set up to determine the best formulation (AF, aluminium hydroxide (Al) suspension, mixed SE or liposome containing the saponin Quillaja saponaria-21 (LSQ)), and the optimal doses of CfaEB, dmLT and SLA upon IM vaccination.

### Definition of the dose ranges

One of the first steps in the DoE approach is to define the range of doses that will be varied during the experiment. The upper bound of CfaEB was set at 10 µg as it was determined that two intradermal immunizations, 3 weeks apart, with 10 µg CfaEB (plus LT(R192G) or LT(R192G/L211A)) in BALB/c mice, reached a plateau in term of serum antigen-specific IgG and immunoglobulin A responses, as well as functional antibodies^53^. The upper bound of the SLA dose was fixed at 5 µg with an optimal dose comprised between 1 and 5 µg depending on both the overall vaccine formulation and the targeted immune response. We chose 0.05 µg as the lower dose bound, since this dose was expected to have minimal adjuvant activity. When administered intradermally with CfaEB, dmLT induced proportional and transient reactogenicity between 0.01 and 1 µg dose, with similar adjuvanticity and antigenicity^53^. Based on those observations, we limited the upper dose of dmLT to 1 µg per dose (Table 3).

**Table 3:** Qualitative and quantitative factors used to build the experimental design.

		Levels			
*Qualitative factor*					
X_1_	Type of formulation:	SE	AF	Alum	LSQ
*Quantitative factors*		*Experimental domain*			
X_2_	CfaEB	0.3–10 µg			
X_3_	SLA	0.05–5 µg			
X_4_	dmLT	0.03–1 µg			

### Experimental strategy

We hypothesized that the dose range of the quantitative factors covers the optimal responses. In other words, the empirical mathematical model postulated to estimate the responses in the whole studied space was a second-order polynomial to include the curvature effects for the doses. Moreover, since we expect that the behaviour of the components depends on the formulation, this model included the interactions between doses and formulations as well (Supplementary Eq. (1)). Therefore, a D-optimal experimental design was built to estimate the 40 coefficients of the model that emerged. Despite classical fractional designs needed currently to be analysed in one block due to missing combinations, this experimental design was selected to allow separate interpretation of the results of each formulation, without losing mathematical relevance. To test the validity of the model, two test points by formulation were added. Finally, the experimental design consisted of 16 groups (14 + 2 test points) per formulations for a total of 64 distinct vaccine preparations (Supplementary Table 5).

### Desirability analysis

Whereas the mathematical model was applied to all the readouts separately, a multicriteria optimization using the desirability function approach was undertaken to determine the ‘compromise zone’ where all experimental responses were satisfactory. For each immune response (*Y*_i_) a desirability function (*d*_i_) was set, transforming the modelled response through a linear regression varying from 0% to 100%. Lower and upper bounds were fixed, respectively, as the first quartile of the 64 observed means and the maximum of the 640 observed individual values for each response, respectively (Table 1, columns B and C). To combine mathematical models of all the nine immune readouts, an overall desirability function (*D*), based on individual desirability functions (*d*_i_) was constructed using weightings according to the targeted immune response. Therefore, the most important property was assigned a weight of 5 and the least important assigned a weight of 1 (Table 1, column D). The mathematical formula is given in Supplementary Eq. (2).

### Statistical analysis

Data were normalized by log10 transformation. To avoid any experimental bias, bioassays were performed for all the groups in duplicate, leading to two balanced experimental blocks. Consequently, means were calculated for each block of five mice, and then the average of the two blocks calculated. The significance of the coefficients of the model was calculated using Student’s *T* test and inter-mice variance. The overall variance was the average of individual inter-mice variances and arbitrarily considered at 30 degrees of freedom. The mathematical model was validated through three different statistical assessments. A Fisher *F* test was performed to assess if the variation of the response could be linked to the experimental conditions. The regression is significant when this *p*-value is < 0.05. Experimental variance was compared to residual variance with variance Fisher *F* test to verify how far the calculated values were from the experimental values (*p*-value expected higher than 0.05). The *R*^2^ calculation estimated the overall variance explained by the model, and the residuals distribution allowed to verify how well the model fitted observed values. The adjusted *R*^2^ was used to consider the number of degrees of freedom and thus provided an unbiased estimate of the population *R*^2^. Finally, test points were used to verify the fitness of the model in complementary experiments, not used to estimate the mathematical model. The differences between the experimental values and the predicted values were statistically tested using the Student’s *T* test (two-sided) and the variance provided by the ANOVA. Analysis of the DoE was performed using the Azurad v.2019 software.

Non-parametric Spearman correlation coefficient (*r*) was determined using GraphPad Prism 7 software (GraphPad Software, Inc., La Jolla, CA, USA). Data from confirmatory studies were analysed using GraphPad Prism 7 software by one-way ANOVA with Tukey’s multiple comparisons post-test (two-sided). Values were considered significantly different with *p* < 0.05 (*), *p* < 0.01 (**), *p* < 0.001 (***), or *p* < 0.0001 (****).

### Ethics statement

All animal experiments and protocols were approved by the Infectious Disease Research Institute’s Institutional Animal Care and Use Committee. All human blood research reported here was reviewed and approved by Western Institutional Review Board and all human subjects underwent Institutional Review Board-approved informed consent process. Heparinized human blood samples were collected from normal, healthy donors using standard phlebotomy techniques.

### Mice, immunizations and tissue harvesting

Female BALB/c mice aged 6–10 weeks (Jackson Laboratory, Bar Harbor, ME, USA) were housed in specific pathogen-free conditions and fed ad libitum.

Ten mice per vaccine composition were immunized twice (prime Day 0 and booster Day 21) via a 50 µL IM injection in the calf muscles of both hind limbs with a specific vaccine composition consisting of a defined dose of CfaEB, dmLT and SLA formulated in: a 2% squalene-in-water stable emulsion (SE), LSQ, an AF, or on Al. CfaEB, dmLT, SLA-SE, SLA-AF, SLA-LSQ, and SLA-Al were produced as previously described^19,22,54^. In total, 64 experimental conditions (divided into two blocks of five mice for each group) were tested. Blood was collected on Day 21 and Day 35, and serum separated using Microvette Z-gel (SARSTEDT AG&Co, Nümbrecht, Germany) and stored at −20 °C until analysis. On Day 35, ~15 cm distal ileum was harvested into Hanks’ balanced salt solution and then washed with Hanks’ balanced salt solution containing 0.6 mM phenylmethylsulfonyl fluoride (Fluka Chemie AG, Buchs, Switzerland) and protease inhibitor cocktail (Sigma P8849; Merck KGaA, Darmstadt, Germany). The liquid was centrifuged at 1660 × *g* at 4 °C for 20 min to pellet faecal material. IW supernatant was then collected and stored at −20 °C until analysis. On Day 35, bone marrow was isolated from both rear femurs and red blood cells were immediately lysed with red blood cell lysis buffer (Invitrogen, Carlsbad, CA, USA).

### Haemagglutination inhibition assay

The HAI assay was carried out as described by Anantha et al.^55^ with some modifications^21^. Briefly, ETEC H10407 (CFA/I+, LT+, STh+, STp+) bacteria lawn grown overnight on CFA plate with 50 µM desferal was harvested and stored at −80 °C in OD_600_ = 20 aliquots. First, the minimal haemagglutination titre (MHT), i.e. the lowest concentration of ETEC H10407 that resulted in agglutination of the red blood cells, was determined. An aliquot of bacteria at OD_600_ = 20 was serially diluted in 96-well plates and incubated in phosphate buffer saline + 0.5% d-mannose, containing 1.5% human red blood cells, at 4 °C on a shaker at 450 rpm for 30 min first and then at 550 rpm for 1 h.

For the inhibition assay, serum or IW samples were serially diluted and mixed to an equal volume of ETEC at four times the MHT and incubated at 37 °C with gentle agitation (on a shaker at 50 rpm) for 30 min. An equal volume of 1.5% human red blood cells was added to each well, followed by incubation at 4 °C on a shaker at 450 rpm for 30 min and then 550 rpm for 1 h. The HAI titre was defined as the reciprocal of the last well to show complete inhibition of haemagglutination by the antibodies present in the sample.

### LT neutralization assay

The LT neutralization assay was carried out as described by Liang et al.^21^ In brief, the optimal toxin dose (EC_10_), i.e. the concentration of LT for which 90% of its maximal effect is observed, was determined. First, 750 cells/15 µL/well of Vero cells (ATCC, #atcc-ccl-81) were plated in 384-well culture plates (PerkinElmer) in IMDM + GlutaMax (Thermo Fisher Scientific) with 4% foetal bovine serum and 1% penicillin–streptomycin and incubated for 18–24 h in 37 °C, 5% CO_2_ incubator. LT was serially diluted in stimulation buffer per Lance Ultra cAMP kit (Perkin Elmer) protocol; 10 µL of each LT dilutions were added to the Vero cells and incubated for 2.5 h in 37 °C, 5% CO_2_ incubator. The wells were emptied and 5 µL of each of Eu-cAMP and uLight-anti-cAMP working solution from Lance Ultra cAMP kit (Perkin Elmer) was added to each well according to manufacturer instructions and incubated at room temperature for 1 h in the dark. The 665/615 nm emission ratio was determined for each well and plotted as a function of LT concentrations. The EC_10_ was calculated using GraphPad Prism.

For the neutralization assay, serial dilutions of serum or IW samples were mixed to full length LT at 2 × EC_10_ concentration (0.05–0.3 ng/µL) at equal volume and incubated at 37 °C for 15 min in a shaker at 100 rpm. The mixture (10 µL) was then added to each well with the Vero cells and incubated for 2.5 h at 37 °C, 5% CO_2_ incubator. The detection reagents Eu-cAMP and uLight-anti-cAMP were added as described above. The 665/615 nm emission ratio was determined for each well and plotted as a function of serum dilution. The IC_50_ was calculated for each sample using GraphPad Prism.

### ELISA IgG antibody titres against the CfaEB and dmLT

The ELISA was carried out as described by Liang et al.^21^ In brief, 384-well plates (Corning 3700) (Thermo Fisher Scientific) were coated with 2 µg/mL of CfaEB antigen or 1 µg/mL dmLT and blocked with phosphate buffer saline (pH 7.1), 0.1% Tween-20, and 1% skimmed milk. Serum or IW samples were diluted and plated over a 12-point series. Detection antibodies conjugated to horse radish peroxidase (SouthernBiotech Birmingham, AL, USA) specific to total IgG were added. Finally, the reaction was developed using the KPL SureBlue (TMB substrate, SeraCare Life Science, Inc., Milford, MA, USA) and stopped using 1 N H_2_SO_4_. OD readings were taken at 450 nm, with data reduction at 570 nm using an automated plate-reader (Biotek Synergy 2, Winooski, VT, USA). The endpoint titres were determined using the dilution at OD_450_ = 0.5.

### ELISpots for bone marrow plasma cells

ASCs were detected in cells harvested from mouse bone marrow using the ELISpot assay. Polyvinylidene fluoride plates with hydrophobic high protein binding immobilon-P membrane (Millipore) (Billerica, MA, USA) were wetted with 35% ethanol, washed three times with phosphate buffer saline, and coated with CfaEB or dmLT (2 µg/mL) in eBiosciences (San Diego, CA, USA) coating buffer at 4 °C overnight. Plates were then washed with phosphate buffer saline-Tween and blocked with Roswell Park Memorial Institute 1640 medium with 10% foetal calf serum for at least 2 h. Bone marrow cells were adjusted to a concentration of 10^7^ cells/mL and four 3-fold serial dilutions were prepared starting with 1 × 10^6^ cells/well in the ELISpot plate and incubated at 37 °C for 5 h. ASCs were detected with anti-mouse IgG H + L (which recognizes heavy and light chains) or immunoglobulin A at 1:1000 (Southern Biotech) in 95% phosphate buffer saline-Tween/5% foetal calf serum. The plates were developed using aminoethyl carbazole solution (Vector Laboratories, Inc., Burlingame, CA, USA) and counted using Cellular Technology Limited ELISpot software (version 2.6.1) (Shaker Heights, OH, USA).

### Reporting summary

Further information on research design is available in the Nature Research Reporting Summary linked to this article.

## Supplementary information

Supplementary Information

Reporting Summary Checklist

## Data Availability

Data are available upon reasonable request from the authors.
