# Supplementary material for: Preclinical optimization of an enterotoxigenic Escherichia coli adjuvanted subunit vaccine using response surface design of experiments
Source: NPJ Vaccines. 2020 Sep 11;5:83. doi: 10.1038/s41541-020-00228-w (PMC7486917; doi:10.1038/s41541-020-00228-w)
Supplement: Supplementary file 1 — Supplementary Information [file 41541_2020_228_MOESM1_ESM.pdf]

Supplemental information

Supplementary Equation 1: Postulated mathematical models associated with each type of variables are:

- Qualitative factor X1: $Y_{qual.} = b_0 + b_{1A} X_{1A} + b_{1B} X_{1B}$
- Quantitative factors X2, X3, X4 : $Y_{quant.} = b_0 + b_2 X_2 + b_3 X_3 + b_4 X_4 + b_{22} X_2^2 + b_{33} X_3^2 + b_{44} X_4^2 + b_{23} X_2X_3 + b_{24} X_2X_4 + b_{34} X_3X_4$

To take into consideration the interaction effect between the qualitative and quantitative factors, a product model was postulated:

$$\mathbf{Y} = Y_{qual} \times Y_{quant.} = b_0 + b_{1A} X_{1A} + b_{1B} X_{1B} + b_{1C} X_{1C} + b_2 X_2 + b_{21A} X_2X_{1A} + b_{21B} X_2X_{1B} + b_{21C} X_2X_{1C} + b_3 X_3 + b_{31A} X_3X_{1A} + b_{31B} X_3X_{1B} + b_{31C} X_3X_{1C} + b_4 X_4 + b_{41A} X_4X_{1A} + b_{41B} X_4X_{1B} + b_{41C} X_4X_{1C} + b_{22} X_2^2 + b_{221A} X_2^2X_{1A} + b_{221B} X_2^2X_{1B} + b_{221C} X_2^2X_{1C} + b_{33} X_3^2 + b_{331A} X_3^2X_{1A} + b_{331B} X_3^2X_{1B} + b_{331C} X_3^2X_{1C} + b_{44} X_4^2 + b_{441A} X_4^2X_{1A} + b_{441B} X_4^2X_{1B} + b_{441C} X_4^2X_{1C} + b_{23} X_2X_3 + b_{231A} X_2X_3X_{1A} + b_{231B} X_2X_3X_{1B} + b_{231C} X_2X_3X_{1C} + b_{24} X_2X_4 + b_{241A} X_2X_4X_{1A} + b_{241B} X_2X_4X_{1B} + b_{241C} X_2X_4X_{1C} + b_{34} X_3X_4 + b_{341A} X_3X_4X_{1A} + b_{341B} X_3X_4X_{1B} + b_{341C} X_3X_4X_{1C}$$

To estimate the 40 coefficients of this model and predict the response in the whole domain with good quality a D-optimal design of experiments, was built from the Fedorov algorithm (Fedorov, V.V. Theory of Optimal Experiments, Academic Press, N.Y., 1972). This design consisted of 56 experiments.

Supplementary Equation 2: Overall desirability function (D)

$$D=\sqrt[25]{d_1^{4}\times d_2^{3}\times d_3^{5}\times d_4^{4}\times d_5^{3}\times d_6^{1}\times d_7^{1}\times d_8^{2}\times d_9^{2}}$$

The optimal condition was multidimensional space defined as the largest volume around the point where the global desirability was maximum, in practice different from 0, with the guarantee that the objectives were satisfied with a probability of 95% (Quality by Design concept)(Sangshetti, J.N. et al. *Arab. J. Chem.* **10**, S3412-S3425 (2017).

Supplementary Table 1: Coefficients for HAI response models

Formulation	SE	SE	AF	AF	AF	LSQ	LSQ	LSQ	AI	AI	AI
Day	35	35	21	35	35	21	35	35	21	35	35
Sample	Serum	IW	Serum	Serum	IW	Serum	Serum	IW	Serum	Serum	IW
Constant	3.51	0.21	2.14	3.62	2.06	2.49	3.72	2.30	2.55	4.09	2.12
CfaEB	0.12	0.55	**-0.15**	-0.05	0.01	**0.24**	0.10	0.04	0.10	**0.15**	0.09
SLA	0.09	0.21	0.00	-0.06	0.03	-0.02	0.02	-0.02	0.01	-0.03	0.06
dmLT	-0.05	0.55	0.03	0.02	-0.05	-0.02	0.00	-0.08	-0.09	-0.04	-0.13
CfaEB * CfaEB	0.05	0.21	0.05	-0.16	0.09	-0.08	0.09	0.08	0.05	0.05	-0.01
SLA * SLA	0.04	0.55	**-0.43**	0.02	0.10	-0.10	-0.14	-0.02	-0.02	-0.09	-0.09
dmLT * dmLT	0.01	0.21	**0.40**	0.11	0.00	0.10	0.07	0.01	-0.09	-0.02	0.16
CfaEB * SLA	-0.02	0.55	0.12	-0.05	0.02	-0.03	-0.04	0.02	-0.02	0.05	-0.02
CfaEB * dmLT	-0.01	0.21	-0.15	0.05	-0.02	0.04	-0.05	-0.03	-0.04	0.03	0.02
SLA * dmLT	0.04	0.55	0.09	-0.02	-0.07	-0.02	0.06	-0.07	0.01	0.00	-0.03
Adjusted R^2	0.39	N/A	0.48	0.17	N/A	0.77	0.45	0.83	0.64	0.83	0.15

Student's T test; Bold: p-value <0.05

Supplementary Table 2: Coefficients for LT neutralization response models

Formulation	SE	AF	AF	LSQ	LSQ	Al	Al
Day	21	21	35	21	35	21	35
Sample	Serum	Serum	Serum	Serum	Serum	Serum	Serum
Constant	1.89	1.63	3.64	1.39	3.08	1.57	3.35
CfaEB	0.03	-0.09	0.05	-0.01	-0.06	0.03	-0.03
SLA	0.13	-0.03	0.01	0.10	0.10	0.00	0.04
dmLT	**0.28**	**-0.21**	0.06	**0.46**	**0.57**	**0.39**	**0.69**
CfaEB * CfaEB	0.19	0.11	-0.20	-0.12	-0.06	-0.11	-0.01
SLA * SLA	0.15	0.02	0.21	0.05	-0.16	0.12	0.04
dmLT * dmLT	-0.11	-0.01	0.02	0.22	0.13	0.00	-0.13
CfaEB * SLA	-0.06	-0.12	0.01	0.02	-0.11	-0.10	-0.10
CfaEB * dmLT	**-0.23**	-0.20	-0.14	0.10	0.09	-0.09	-0.01
SLA * dmLT	0.00	-0.02	0.06	0.08	0.05	0.03	0.03
Adjusted R^2	0.88	0.11	n.d.	0.96	0.98	0.65	0.96

Student's T test; Bold: p-value <0.05

Supplementary Table 3: Coefficients for IW IgG response models

Formulation	SE	AF	AF	LSQ	LSQ	Al	Al
Antigen	CfaEB	dmLT	CfaEB	dmLT	CfaEB	dmLT	CfaEB
Sample	IW	IW	IW	IW	IW	IW	IW
Constant	3.37	3.24	3.70	3.88	4.31	2.93	3.93
CfaEB	0.07	-0.04	0.09	-0.02	0.13	-0.03	**0.20**
SLA	0.00	0.03	**0.20**	0.12	0.09	0.08	0.03
dmLT	0.00	**0.25**	-0.15	0.38	0.10	**0.46**	-0.07
CfaEB * CfaEB	0.06	-0.01	-0.14	0.10	0.04	0.04	-0.01
SLA * SLA	0.11	0.09	0.33	-0.14	-0.15	-0.14	-0.09
dmLT * dmLT	-0.13	-0.04	-0.11	0.01	-0.06	-0.08	-0.01
CfaEB * SLA	0.01	0.04	-0.02	-0.05	-0.04	0.02	0.05
CfaEB * dmLT	0.00	-0.05	0.06	-0.02	0.01	-0.03	-0.01
SLA * dmLT	0.05	0.02	0.01	-0.13	-0.14	0.03	0.12
Adjusted R^2	n.d.	0.65	0.82	0.92	0.77	0.86	0.36

Student's T test; Bold: p-value <0.05

Supplementary Table 4: Coefficients for ASC response models

Formulation	SE	AF	AF	LSQ	LSQ	AI	AI
Antigen	dmLT	dmLT	CfaEB	dmLT	CfaEB	dmLT	CfaEB
Sample	ASC	ASC	ASC	ASC	ASC	ASC	ASC
Constant	1.57	1.18	1.97	1.69	2.33	1.20	1.93
CfaEB	-0.02	0.00	0.06	-0.04	-0.07	0.04	0.13
SLA	0.05	0.03	0.00	-0.02	0.11	0.07	-0.09
dmLT	**0.39**	**0.37**	**0.27**	**0.26**	**0.19**	**0.35**	**0.22**
CfaEB * CfaEB	-0.11	-0.23	0.01	-0.03	-0.09	0.13	0.09
SLA * SLA	-0.03	0.21	0.13	-0.10	-0.06	-0.02	-0.02
dmLT * dmLT	-0.06	0.28	0.10	-0.04	-0.06	-0.08	-0.01
CfaEB * SLA	-0.03	-0.04	-0.03	0.12	0.07	-0.02	-0.05
CfaEB * dmLT	0.03	-0.04	0.00	0.00	0.03	0.01	-0.01
SLA * dmLT	-0.01	0.00	0.04	0.09	-0.06	-0.11	0.03
Adjusted R^2	0.79	0.48	n.d.	0.47	0.58	0.89	0.60

Student's T test; Bold: p-value <0.05

Supplementary Table 5: Design of experiments used to establish the RSM for each immune response

Group	CfaEB (µg)	SLA (µg)	dmLT (µg)	Formulation
1	0.3	0.05	0.03	SE
2	10	0.05	0.03	SE
3	1.7	0.5	0.03	SE
4	0.3	5	0.03	SE
5	10	5	0.03	SE
6	1.7	0.05	0.17	SE
7	0.3	0.5	0.17	SE
8	10	0.5	0.17	SE
9	1.7	5	0.17	SE
10	0.3	0.05	1	SE
11	10	0.05	1	SE
12	1.7	0.5	1	SE
13	0.3	5	1	SE
14	10	5	1	SE
15*	1.7	0.5	0.17	SE
16*	1	0.1	1	SE
17	0.3	0.05	0.03	AF
18	10	0.05	0.03	AF
19	1.7	0.5	0.03	AF
20	0.3	5	0.03	AF

21	10	5	0.03	AF
22	0.3	0.05	0.17	AF
23	10	0.5	0.17	AF
24	1.7	5	0.17	AF
25	0.3	0.05	1	AF
26	1.7	0.05	1	AF
27	10	0.05	1	AF
28	0.3	0.5	1	AF
29	0.3	5	1	AF
30	10	5	1	AF
31*	1.7	0.5	0.17	AF
32*	4.2	0.2	0.4	AF
33	0.3	0.05	0.03	LSQ
34	10	0.05	0.03	LSQ
35	10	0.5	0.03	LSQ
36	0.3	5	0.03	LSQ
37	1.7	5	0.03	LSQ
38	10	5	0.03	LSQ
39	1.7	0.05	0.17	LSQ
40	0.3	0.5	0.17	LSQ
41	10	5	0.17	LSQ
42	0.3	0.05	1	LSQ
43	10	0.05	1	LSQ
44	1.7	0.5	1	LSQ
45	0.3	5	1	LSQ
46	10	5	1	LSQ
47*	1.7	0.5	0.17	LSQ
48*	0.7	3	0.7	LSQ
49	0.3	0.05	0.03	Alum
50	10	0.05	0.03	Alum
51	1.7	0.5	0.03	Alum
52	0.3	5	0.03	Alum
53	10	5	0.03	Alum
54	1.7	0.05	0.17	Alum
55	0.3	0.5	0.17	Alum
56	10	0.5	0.17	Alum
57	1.7	5	0.17	Alum
58	0.3	0.05	1	Alum
59	10	0.05	1	Alum
60	1.7	0.5	1	Alum
61	0.3	5	1	Alum
62	10	5	1	Alum
63*	1.7	0.5	0.17	Alum

64*	1	0.1	1	Alum

* test points

Glossary / Definition

AF	aqueous formulation
Al	aluminium oxyhydroxide
ASC	antibody secreting cells
cAMP	cyclic adenosine monophosphate
CF	colonization factor
CS	coli surface
CFA/I	colonization factor antigen I
CfaEB	colonization factor antigen fimbrial subunit E and B fusion
dmLT	double mutated heat-labile toxin
DoE	design of experiment
ETEC	enterotoxigenic *Escherichia coli*
HAI	haemagglutination
IgG	immunoglobulin G
IM	intramuscular
IW	intestinal washes
LSQ	liposomal Quillaja saponaria-21
LT	heat-labile toxin
SE	stable emulsion
SLA	second-generation lipid adjuvant
ST	stable toxin
TLR	Toll-like receptor
TLR4	Toll-like receptor 4 ligand
Linear effect	the effect of a factor is linear when the response evolves in a rectilinear way with regard to this factor

Square effect	the effect of a factor is squared when the response evolves in a parabolic way (that is "shape of bell") when this factor evolves
Interaction	we speak about interaction between two factors when the effect on the response related to one of the factors depends on the level of the other factor
Significance:	the significance of the statistical tests was estimated at a 5 % level (a P-value lower than 5 % or 0.05 corresponds to a significant test)
