# Supplementary material for: Preclinical optimization of an enterotoxigenic Escherichia coli adjuvanted subunit vaccine using response surface design of experiments
Source: NPJ Vaccines. 2020 Sep 11;5:83. doi: 10.1038/s41541-020-00228-w (PMC7486917; doi:10.1038/s41541-020-00228-w)
Supplement: Supplementary file 2 — Reporting Summary Checklist [file 41541_2020_228_MOESM2_ESM.pdf]

nature research

Corresponding author(s): David Poncet or Catherine Hessler

Last updated by author(s): May 14, 2020

Reporting Summary

Nature Research wishes to improve the reproducibility of the work that we publish. This form provides structure for consistency and transparency in reporting. For further information on Nature Research policies, see our Editorial Policies and the Editorial Policy Checklist.

Statistics

For all statistical analyses, confirm that the following items are present in the figure legend, table legend, main text, or Methods section.

n/a	Confirmed	
☐	☒	The exact sample size (*n*) for each experimental group/condition, given as a discrete number and unit of measurement
☐	☒	A statement on whether measurements were taken from distinct samples or whether the same sample was measured repeatedly
☐	☒	The statistical test(s) used AND whether they are one- or two-sided<br>*Only common tests should be described solely by name; describe more complex techniques in the Methods section.*
☒	☐	A description of all covariates tested
☐	☒	A description of any assumptions or corrections, such as tests of normality and adjustment for multiple comparisons
☐	☒	A full description of the statistical parameters including central tendency (e.g. means) or other basic estimates (e.g. regression coefficient) AND variation (e.g. standard deviation) or associated estimates of uncertainty (e.g. confidence intervals)
☐	☒	For null hypothesis testing, the test statistic (e.g. *F*, *t*, *r*) with confidence intervals, effect sizes, degrees of freedom and *P* value noted<br>*Give P values as exact values whenever suitable.*
☒	☐	For Bayesian analysis, information on the choice of priors and Markov chain Monte Carlo settings
☒	☐	For hierarchical and complex designs, identification of the appropriate level for tests and full reporting of outcomes
☒	☐	Estimates of effect sizes (e.g. Cohen's *d*, Pearson's *r*), indicating how they were calculated

Our web collection on statistics for biologists contains articles on many of the points above.

Software and code

Policy information about availability of computer code

Data collection	ELISA plates were read using PerkinElmer VictorX4 / Elispot: Cellular Technology Limited ELISpot software (version 2.6.1) (Shaker Heights, OH, USA)
Data analysis	ELISA, Elispot, LT neut, Haemagglutination inhibition assay (HAI): data were exported as Excel files and analyzed using GraphPad Prism 7 Software (GraphPad Software, Inc., La Jolla, CA, USA); Analysis of the DoE was performed using the Azurad v.2019 software.

For manuscripts utilizing custom algorithms or software that are central to the research but not yet described in published literature, software must be made available to editors and reviewers. We strongly encourage code deposition in a community repository (e.g. GitHub). See the Nature Research guidelines for submitting code & software for further information.

Data

Policy information about availability of data

All manuscripts must include a data availability statement. This statement should provide the following information, where applicable:

- Accession codes, unique identifiers, or web links for publicly available datasets
- A list of figures that have associated raw data
- A description of any restrictions on data availability

The data that support the findings of this study are available from the corresponding author upon reasonable request.

Field-specific reporting

Please select the one below that is the best fit for your research. If you are not sure, read the appropriate sections before making your selection.

☒ Life sciences ☐ Behavioural & social sciences ☐ Ecological, evolutionary & environmental sciences

For a reference copy of the document with all sections, see nature.com/documents/nr-reporting-summary-flat.pdf

Life sciences study design

All studies must disclose on these points even when the disclosure is negative.

Sample size	The number of groups for the DOE has been determined by using Azurad v.2019 software. The sample size per group was determinded based on what is currently done for this kind of study for a sufficient statistical power
Data exclusions	No data were excluded
Replication	The findings from the mathematical model were experimentally confirmed for SE and LSQ formulations in a subsequent mouse study
Randomization	NA
Blinding	NA

Reporting for specific materials, systems and methods

We require information from authors about some types of materials, experimental systems and methods used in many studies. Here, indicate whether each material, system or method listed is relevant to your study. If you are not sure if a list item applies to your research, read the appropriate section before selecting a response.

Materials & experimental systems

n/a	Involved in the study	
☒	☐	Antibodies
☒	☐	Eukaryotic cell lines
☒	☐	Palaeontology and archaeology
☐	☒	Animals and other organisms
☒	☐	Human research participants
☒	☐	Clinical data
☒	☐	Dual use research of concern

Methods

n/a	Involved in the study	
☒	☐	ChIP-seq
☒	☐	Flow cytometry
☒	☐	MRI-based neuroimaging

Animals and other organisms

Policy information about studies involving animals; ARRIVE guidelines recommended for reporting animal research

Laboratory animals	Female BALB/c mice aged 6–10 weeks (Jackson Laboratory, Bar Harbor, ME, USA) were housed in specific pathogen free conditions
Wild animals	The study did not involve wild animals
Field-collected samples	The study did not involve samples collected in the field
Ethics oversight	All animal experiments and protocols were approved by the Infectious Disease Research Institute's Institutional Animal Care and Use Committee (616 Eastlake Ave E, Ste 400, Seattle, WA 98102, USA)

Note that full information on the approval of the study protocol must also be provided in the manuscript.
